# Socioemotional Features and Resilience in Italian University Students with and without Dyslexia

**DOI:** 10.3389/fpsyg.2016.00478

**Published:** 2016-03-31

**Authors:** Marta Ghisi, Gioia Bottesi, Anna M. Re, Silvia Cerea, Irene C. Mammarella

**Affiliations:** ^1^Department of General Psychology, University of PadovaPadova, Italy; ^2^Department of Developmental and Social Psychology, University of PadovaPadova, Italy

**Keywords:** Dyslexia, Socioemotional features, University students, Italian sample, DSM-5

## Abstract

Dyslexia is a permanent condition characterized by reading difficulties that include inaccurate or slow and effortful word reading, poor decoding, and poor spelling abilities. People with dyslexia may have psychological and psychopathological issues such as low self-esteem, poor resilience, and symptoms of depression and anxiety. They may also develop social problems and emotional issues, as well as low academic and social self-efficacy. The present study aimed to assess the psychological features of a sample of 28 Italian university students with dyslexia, comparing them with a control group of typically developing students matched for gender, education, and academic discipline, to enhance our knowledge of dyslexia outcomes in an Italian setting. The results show that university students with dyslexia experience higher levels of somatic complaints, social and attentional problems, lower self-esteem, and higher depression scores than controls, while no difference emerged between the two groups’ resilience scores. In conclusion, the present findings suggest that university students with dyslexia report more psychological issues than students without dyslexia and could benefit from intervention to improve their psychological and physical well-being.

## Introduction

The term “dyslexia” refers to a pattern of learning difficulties consisting in inaccurate or slow and effortful word reading, with weaknesses in decoding and spelling. It is a specific learning disorder, classified among the neurodevelopmental disorders in the Fifth edition of the Diagnostic and Statistical Manual of Mental Disorders (DSM-5; American Psychiatric Association, 2014).

Despite recent evidence of improvements in phonological awareness (i.e., an individual’s understanding of the phonological structure of words) occurring in adolescence and early adulthood, dyslexia is a permanent condition and reading abilities remain impaired at all ages (Kemp et al., 2009; Campanini et al., 2010). Even individuals showing improvements as they grow older continue to take longer to complete reading and writing tasks in academic or professional situations (Hatcher et al., 2002). It has been demonstrated that this affects the psychological features of adolescents and young adults with dyslexia, sometimes resulting in various forms of psychopathology (Mugnaini et al., 2009). In fact, dyslexia has been associated with psychological disorders (Undheim, 2003), and the more severe the dyslexia, the higher the level of the associated psychological symptoms. Comorbidities such as dyscalculia, dysorthographia, and attention-deficit/hyperactivity disorder (ADHD) also correlate with more severe psychological impairments (Martínez and Semrud-Clikeman, 2004; Mugnaini et al., 2009).

The literature highlights that adolescents with dyslexia are more likely to withdraw from school (Daniel et al., 2006), develop social problems (Sabornie, 1994; Wiener and Schneider, 2002), be emotionally disturbed (Capozzi et al., 2007), and suffer from low academic and social self-efficacy, low mood, and loss of hope and motivation when dealing with scholastic tasks (Lackaye and Margalit, 2006). While studies on dyslexic children and adolescents have reported social isolation, behavioral issues (particularly disruptive and aggressive behavior), and emotional problems (Sabornie, 1994; Boetsch et al., 1996; Rutter, 2000; Lyytinen et al., 2001; Twenge et al., 2001), only a small number of studies to date have investigated these issues in samples of young or older adults, especially in Italy. The available findings, obtained from self-report questionnaires, indicate that dyslexia negatively affects emotional security, daily life, and work productivity. In particular, adults with dyslexia have reported fear of failure, frustration, low self-confidence, difficulties in intimate relationships, and a paucity of friends (Hellendoorn and Ruijssenaars, 2000; McNulty, 2003).

In the light of evidence of a relationship between academic difficulties and psychological problems (Drum et al., 2009), university students with dyslexia are likely to be at particular risk of mental health problems. Gregg et al. (1992) found that university students with dyslexia reported anxiety and depression and Riddick et al. (1999) found that students with dyslexia experienced more feelings of academic ineptitude than other students. Negative emotions and anxiety related to dyslexia have been experienced both during compulsory schooling and at university. In another study (Carroll and Iles, 2006), university students with dyslexia reported high trait and state anxiety in situations where their reading abilities were tested. These findings suggest that having to read routinely might be an important stressor and source of anxiety for individuals with dyslexia, particularly in situations requiring accuracy in reading and writing, as commonly encountered at university (Carroll and Iles, 2006). On the other hand, some studies found no differences between university students with dyslexia and without dyslexia in terms of their symptoms of anxiety and depression (Hoy et al., 1997; Mattek and Wierzbicki, 1998; Riddick et al., 1999; Nelson and Gregg, 2012).

Such inconsistent data prompted further research in an effort to identify putative psychological protective factors in individuals with dyslexia (Cosden, 2001; Sorensen et al., 2003; Margalit, 2004). The main factors that emerged as influencing a good psychological adjustment in this population were: early diagnosis (Ingesson, 2007; Pizzoli et al., 2011); religious identity (Svetaz et al., 2000); support within the family and at school (Scott and Scherman, 1992; Hellendoorn and Ruijssenaars, 2000; Al-Yagon and Mikulincer, 2004; Stampoltzis and Polychronopoulou, 2009); and awareness of the individual’s reading difficulties exhibited by parents, teachers, and the individuals with dyslexia themselves (Cosden et al., 1999; Al-Yagon and Mikulincer, 2004). Importantly, a thorough understanding of the problem promotes the setting of realistic scholastic goals for children with dyslexia, and consequently to their successful achievement, thereby fostering a positive self-concept and improving self-esteem (Heyman, 1990; Rothman and Cosden, 1995; Burden and Burdett, 2007). In a recent Italian study (Pizzoli et al., 2011), young adults whose dyslexia had been diagnosed late (in adulthood) consistently revealed negative affects, such as a sense of shame and incompetence, whereas 48% of those who had been diagnosed during primary school reported having no problems, or that their parents and/or friends supported them adequately, and this enabled them to cope with their dyslexia. Another potentially protective factor is resilience, which was found negatively correlated with the number of life and academic difficulties reported by adults with dyslexia completing a university degree or after graduating (Stack-Cutler et al., 2014).

Given the dearth of research on the psychological features of university students with dyslexia (especially in Italy), the present study was designed to assess these characteristics in early-diagnosed dyslexic Italian undergraduates. It is worth noting that a cross-national comparison of the prevalence of dyslexia in Italy and the United States indicated that the conditions is more common in the United States than in Italy, even considering that the use of different methods and diagnostic criteria leads to different figures for the prevalence of dyslexia (ranging from 3.6 to 8.5% in Italy as opposed to 4.5 to 12.0% in the United States) (Lindgren et al., 1985). Barbiero et al. (2012) recently estimated the prevalence of dyslexia in Italy at 3.1 or 3.2%, depending on the criteria adopted.

Although the literature indicates that dyslexia is pretty much incident in countries characterized by orthographically deep (e.g., English) as opposed to shallow (e.g., Italian) languages, one is not allowed to argue that the socioemotional consequences of dyslexia are likely to be more severe in the former than in the latter. The inconsistent data on the socioemotional features of deep language-speaking university students with dyslexia (Gregg et al., 1992; Hoy et al., 1997; Mattek and Wierzbicki, 1998; Riddick et al., 1999; Carroll and Iles, 2006; Nelson and Gregg, 2012) certainly cannot support any precise hypotheses on those of Italian university students with dyslexia. In addition, the potential differences between countries like Italy, the United States, and the United Kingdom in the type of specialist provision for pupils with dyslexia in schools and universities make it even more difficult to advance specific hypotheses. Despite making directional predictions of the likely outcomes could be problematic, some logical ones can be traced given main literature findings on young adolescents with dyslexia. Indeed, evidence suggest that young adults with dyslexia could be particularly prone to develop social problems and behavioral issues (Sabornie, 1994; Boetsch et al., 1996; Rutter, 2000; Lyytinen et al., 2001; Twenge et al., 2001; Wiener and Schneider, 2002), as well as emotional disturbances (Lackaye and Margalit, 2006; Capozzi et al., 2007). Furthermore, undergraduate students with dyslexia have been observed to show low levels of self-esteem and high levels of anxiety and negative emotions (Gregg et al., 1992; Riddick et al., 1999; Carroll and Iles, 2006). Therefore, we expected to observe lower levels of self-esteem, higher levels of anxious and depressive symptoms, and more social problems in a sample of Italian undergraduate students with dyslexia compared to a sample of Italian undergraduate students without dyslexia. Since our sample of participants with dyslexia was made up of individuals who did not withdraw from school, but rather decided to attend university courses, we did not expect the two samples to differ in regard to resilience.

## Materials and Methods

### Participants

The dyslexia group comprised 28 students with dyslexia (20 males and 8 females; mean age = 20.61 years, *SD* = 1.62) attending their first year of university studies in the following disciplines: Engineering (*N* = 7), Agriculture (*N* = 6), Psychology (*N* = 3), Food Science (*N* = 3), Political Sciences (*N* = 2), Education Sciences (*N* = 2), Pharmacy (*N* = 1), Law (*N* = 1), Economics (*N* = 1), Statistics (*N* = 1), and Natural Sciences (*N* = 1). They had all been diagnosed with dyslexia during their primary school years by specialist services forming part of the Italian National Health System. Some of these students had received specific training during their school years. Their diagnosis met the requirements of the DSM-IV (American Psychiatric Association, 2000) and complied with the guidelines typically adopted by public services and recently shared in an official document (see also Consensus Conference, 2007), namely: a normal level of general intelligence (IQ above 85); reading performance problems at a clinical level (reading decoding below the fifth percentile); and no neurological, sensory, or educational deficits (e.g., they had not been absent from school for long periods due to poor health) that could have caused their reading impairment. Students with comorbid ADHD were excluded. Although all the participants with dyslexia had been diagnosed during their childhood, they were assessed again at a specialized university center to confirm their diagnosis. In fact, these students had voluntarily contacted the university center that provides specific services for students with learning disabilities to seek help for their dyslexia. Both the learning tasks and the self-report questionnaires (assessing psychological features relevant to this population) used in the present research were routinely administered by the university center to assess all new students reportedly suffering from dyslexia. None of the students receive any specific help for their dyslexia during the assessment phase.

The students with dyslexia were compared with a control group of 28 university students (20 males and 8 females; mean age = 19.61, *SD* = 0.79), matched for gender, education, and academic discipline, who volunteered for the study. The two groups differed slightly in age (the dyslexic group having a slightly higher mean age and larger SD), probably because some of the students with dyslexia did not enroll at university immediately after completing their secondary school education.

The two groups of students (with and without dyslexia) differed in all the tasks administered to measure their reading and writing abilities, the students with dyslexia performing less well than the controls (with large effect sizes, $\eta_{p}^{2}$ > 0.14), while no differences emerged between the groups in the comprehension task, as shown in **Table 1**.

**Table 1:** Differences between students with dyslexia and typical development ones in reading and writing tasks.

	Dyslexic group *M* (*SD*)	Control group *M* (*SD*)	*F*(1,54)	*p*	*$\eta_{p}^{2}$*
Reading text syllable/sec.	4.09 (1.10)	5.97 (1.47)	27.02	<0.001	0.35
Reading text errors	10.38 (6.72)	1.37 (0.94)	47.55	<0.001	0.49
Reading words syllable/sec.	3.28 (1.12)	5.67 (0.80)	77.17	<0.001	0.62
Reading words errors	2.91 (2.56)	0.18 (0.48)	29.59	<0.001	0.38
Non-words syllable/sec.	1.90 (0.84)	3.57 (0.72)	57.32	<0.001	0.54
Non-words errors	5.78 (4.60)	1.00 (1.33)	26.64	<0.001	0.36
Reading Comprehension	15.24 (1.83)	16.22 (1.93)	3.53	0.070	0.06
Dictation errors	0.70 (1.17)	0 (0)	10.12	0.760	0.16
Dictation with articulator suppression errors	9.68 (6.82)	0.30 (0.61)	50.71	0.002	0.50


### Measures

#### Learning Tasks

The measures employed were based on tasks typically used in Italy to assess children with dyslexia, after adapting them for the purpose of assessing older individuals, whereas the tasks under articulatory suppression were devised specifically for assessing university students (Cornoldi et al., 2010; Re et al., 2011). The battery included four tasks recommended in the Italian guidelines for assessing dyslexia (Consensus Conference, 2007), i.e., three reading decoding tests (reading texts, words, and non-words), and a passage-reading comprehension task.

#### Reading Tasks.

##### Text reading

Speed of text reading and errors were assessed with the MT battery (Cornoldi et al., 2010), which is the tool most commonly used in Italy to measuring passage-reading speed and errors, and it has a high test–retest reliability (*r* = 0.97 for reading, and *r* = 0.86 for errors). It comprises different passages for the various levels of difficulty, which increase in terms of the number of syllables and the complexity of the text concerned. The most complex passage (designed for 10th-graders) was used, which is quite long (1,287 syllables) and quite difficult to read because it contains some uncommon technical words. Participants were asked to read the passage aloud, paying attention to their accuracy and speed (the instructions were: “Read as accurately and rapidly as you can”). Reading speed was calculated by dividing the number of syllables of the passage by the time (in seconds) taken to read it. Errors corresponded to the number of words read incorrectly.

##### Word reading

This task is a subtest of a battery for assessing developmental dyslexia and dysorthographia (Sartori et al., 2007) that includes five subtests for assessing various aspects of reading, and three for assessing writing. The battery has only a medium reliability (the mean test–retest coefficients are *r* = 0.77 for speed, and *r* = 0.56 for errors), but it has been validated in a number of studies and included among the recommended tests for assessing reading in Italian (Consensus Conference, 2007). Participants were asked to read four lists of isolated words aloud as accurately and rapidly as possible. The material varied in frequency and concreteness, starting with a list of very common and concrete words, followed by lists of words of decreasing usage frequency and concreteness. Reading speed was calculated by dividing the number of syllables read by the time (in seconds) taken to read them. Errors corresponded to the number of words read incorrectly.

##### Non-word reading

This task is another subtest of the above-mentioned battery for assessing developmental dyslexia and dysorthographia (Sartori et al., 2007). As in the previous task, participants were asked to read the material aloud and as accurately and rapidly as possible. Here again, reading speed was calculated by dividing the number of syllables read by the time (in seconds) taken to read them. Errors corresponded to the number of words read incorrectly.

##### Text comprehension

This task was also derived from the material for 10th-graders in the MT battery (Cornoldi et al., 2010). The test was administered exactly according to the standard procedure used in all standardized Italian reading comprehension tasks, which focus mainly on the student’s ability to find appropriate information in the text in order to answer a series of comprehension questions – i.e., in order to assess the respondent’s comprehension irrespective of any contribution of decoding and text recall (Cornoldi and Oakhill, 1996). Participants were asked to read two passages silently and then answer 20 questions related to the text (10 for each passage). There was no time limit for completion of the task and respondents were told that the time they took was not considered in any way, and that they were allowed to consult the text.

#### Writing Tasks

##### Word dictation

This task consisted of a dictation under two different conditions: normal and with articulatory suppression. The material included two lists of 24 words each; all the words contained three or four syllables, were equally common in normal usage, and posed no particular spelling difficulties (Re et al., 2011). The experimenter dictated at a constant rate of one word every 3 s. In the condition with articulatory suppression, the task was exactly the same, but participants were asked to repeat the syllable “la” aloud continuously during the dictation. A preliminary trial was run to ensure that respondents were perfectly capable of understanding and writing down the dictated words even while articulating. The score corresponded to the number of words written incorrectly.

#### Self-report Measures

The following self-report measures were administered.

The *Connor Davidson Resilience Scale* (CD-RISC; Connor and Davidson, 2003) assesses resilience and the ability to cope with adversities. The CD-RISC contains 25 items rated on a Likert scale from 0 = “not true at all” to 4 = “true nearly all the time” The total scores range from 0 to 100, with higher scores reflecting greater resilience. The original psychometric properties were good; the internal consistency in a community sample was α = 0.89, and the test–retest reliability was *r* = 0.87. Since no Italian version was available, an *ad hoc* translation was prepared adopting the conventional forward–backward procedure (Brislin, 1986).

The *Rosenberg Self-Esteem Scale* (RSES; Rosenberg, 1965); Italian version by (Prezza et al., 1997) is a 10-item self-report scale measuring global self-esteem. Items are rated on a four-point Likert scale (1 = “strongly disagree;” 4 = “strongly agree”). For the original RSES, an internal consistency ranging between α = 0.72 and α = 0.88 has been reported (Gray-Little et al., 1997). The Italian version of the RSES has shown good psychometric properties; its internal consistency was α = 0.84, and the test–retest reliability after 15 days was *r* = 0.76 (Prezza et al., 1997).

The *Beck Depression Inventory-II* (BDI-II; Beck et al., 1996); Italian version by (Ghisi et al., 2006) is a 21-item self-report questionnaire that assesses the severity of the affective, cognitive, motivational, vegetative, and psychomotor components of depression. The BDI-II has shown a very good internal consistency (α = 0.92 among outpatients, and α = 0.93 among college students; Beck et al., 1996). When the Italian version of the BDI-II was administered to 733 undergraduates, 354 community controls, and 135 depressed patients, it revealed excellent psychometric properties (Sica and Ghisi, 2007). The internal consistency was good (α = 0.80), and so was the test–retest reliability after 30 days (*r* = 0.76).

The *Child Behavior Checklist - Youth Self-Report* (CBCL-YSR; Achenbach and Rescorla, 2001); Italian version translated by Frigerio et al. (2001) is composed of 112 items measuring emotional and behavioral problems in children and adolescents. Youths are asked to rate their behavior on a three-point Likert scale ranging from 0 = “not true” to 2 = “very true or often true.” The CBCL-YSR includes eight scales, or “syndromes:” Withdrawn; Somatic Complaints; Anxious/Depressed; Social Problems; Thought Problems; Attention Problems; Rule-Breaking Behavior; and Aggressive Behavior. The scale demonstrated good psychometric properties; its internal consistency ranged from α = 0.71 to α = 0.86, and its test–retest reliability after 8 days ranged between *r* = 0.67 and *r* = 0.88 (Achenbach and Rescorla, 2001).

### Statistical Analyses

Univariate analyses of variance (ANOVAs) were run to compare the groups’ performance in the learning tasks and their scores in the self-report questionnaires. Conventional significance levels were adopted for the CD-RISC, the RSES, and the BDI-II (*p* < 0.05), whereas Bonferroni’s correction for multiple comparisons was applied to the CBCL-YSR subscales (*p* < 0.006). Partial η^2^ were computed to estimate the effect sizes. According to Cohen (1988), η^2^ = 0.01 corresponds to a small effect size, η^2^ = 0.06 to a medium effect size, and η^2^ = 0.14 to a large effect size. All statistical analyses were performed using the Statistical Package for the Social Sciences (SPSS) version 21.

#### Procedure

All participants were assessed individually in a dedicated room, away from sources of noise or other distractions. They were assessed by psychologists specialized in the assessment and treatment of learning disabilities and emotional disorders. The assessment took around 2 h to complete. All participants were administered the learning tasks and then completed a background information chart and four self-report measures, which were administered in counterbalanced order to control for order effects. All the measures used are commonly regarded as non-invasive procedures. If any psychological problems (in either group) or learning difficulties (in the control group) emerged from the assessment, the students concerned were offered intervention at specialized university centers free of charge.

The study was conducted in accordance with the Declaration of Helsinki. All participants entered the study of their own free will and provided their informed consent before taking part. They were informed in detail about the aims of the study, the voluntary nature of their participation, and their right to withdraw from the study at any time and without being penalized in any way. Given the above-mentioned conditions, this project was not submitted to an ethical committee for approval.

## Results

### Differences in Socioemotional Features between the Groups

The ANOVAs revealed that the dyslexic group’s scores on the RSES [*F*(1,54) = 5.26; *p* = 0.026] were lower than those of the control group, whereas no differences emerged for the CD-RISC. The dyslexic group scored higher than controls on the BDI-II [*F*(1,54) = 3.97; *p* = 0.049] and on the subscales concerning Somatic Complaints [*F*(1,54) = 4.39; *p* = 0.041], Social Problems [*F*(1,54) = 8.49; *p* = 0.005], and Attention Problems [*F*(1,54) = 9.55; *p* = 0.003] in the CBCL-YSR. After applying Bonferroni’s correction for multiple comparisons, the CBCL-YSR scale for Somatic Complaints was no longer significant (*p* > 0.006), though a medium effect size was apparent. Medium effect sizes also emerged for the RSES and the BDI-II, whereas the CBCL-YSR scales for Social and Attention Problems showed large effect sizes. It is worth noting that 7 of the 28 students with dyslexia scored above the Italian cutoff on the BDI-II, and that 4 of the 28 students with dyslexia had clinical scores for low self-esteem (*z* > -1.64) on the RSES.

The dyslexic and control groups had similar scores on the CBCL-YSR scales for Withdrawn, Anxious/Depressed, Thought Problems, Rule-Breaking Behavior, and Aggressive Behavior (see **Table 2**).

**Table 2:** Differences between students with dyslexia and typical development ones in self-report measures.

Self-report measures	Dyslexic group *M* (*SD*)	Control group *M* (*SD*)	*F*(1,54)	*p*	*$\eta_{p}^{2}$*
CD-RISC	62.96 (13.60)	64.82 (10.20)	0.33	0.570	0.01
RSES	28.28 (6.17)	31.86 (5.45)	5.26	0.026^∗^	0.09
BDI-II	8.68 (6.99)	5.64 (4.02)	3.97	0.049^∗^	0.07
CBCL-YSR – Withdrawn	4.18 (3.09)	2.96 (2.54)	2.57	0.110	0.04
CBCL-YSR – Somatic Complaints	2.86 (2.76)	1.53 (1.87)	4.39	0.041^∗^	0.08
CBCL-YSR – Anxious/Depressed	7.71 (5.76)	6.64 (3.95)	0.66	0.420	0.01
CBCL-YSR – Social Problems	3.18 (2.05)	1.78 (1.47)	8.49	0.005^∗∗^	0.14
CBCL-YSR – Thought Problems	1.46 (1.55)	1.61 (1.89)	0.09	0.760	0.00
CBCL- YSR – Attention Problems	6.96 (2.91)	4.78 (2.33)	9.55	0.003^∗∗^	0.15
CBCL- YSR – Rule-Breaking Behavior	2.11 (1.50)	2.14 (1.84)	0.01	0.940	0.00
CBCL- YSR – Aggressive Behavior	8.21 (4.28)	7.32 (4.08)	0.64	0.430	0.01


*^∗^ = *p* < 0.05; ^∗∗^ = *p* < 0.006 (for the CBCL-YSR subscales Bonferroni correction for multiple comparisons was applied). CD-RISC, Connor Davidson-Resilience Scale; RSES, Rosenberg Self-Esteem Scale; BDI-II, Beck Depression Inventory-Second Edition; CBCL-YSR, Child Behavior Checklist-Young Self-Report.*

## Discussion

University students with dyslexia have been inadequately investigated to date, especially in Italy. A few previous studies have nonetheless demonstrated that this permanent disorder can cause undergraduates and other adults a number of difficulties when they have to cope with tasks and activities that involve reading and writing (Hanley, 1997; Shaywitz et al., 1999; Hatcher et al., 2002; Lami et al., 2008; Kemp et al., 2009; Singleton et al., 2009). There are reports in the literature of university students with dyslexia having lower self-esteem and resilience, and more psychopathological issues than typically developing undergraduates (Gregg et al., 1992; Riddick et al., 1999; Undheim, 2003; Carroll and Iles, 2006). The main goal of the present study was to assess the psychological features of a group of Italian university students with dyslexia, comparing them with a matched control group.

Overall, our results are consistent with the existing literature thus substantially confirming our hypotheses. The dyslexic group reported significantly lower levels of self-esteem than the controls. University students with dyslexia may encounter several difficulties when their academic work demands good reading and writing skills, and this can generate a negative self-perception when they compare their performance with that of their peers with dyslexia, thus fostering low levels of self-esteem and self-confidence (Riddick et al., 1999; Humphrey, 2002). A low self-esteem can give rise to unpleasant feelings and emotions, sometimes leading to the onset of depressive symptoms such as lack of interest and energy, depressed mood, pessimism, sadness, self-blame, and sleeping and eating disorders. The students with dyslexia involved in the present study also reported higher levels of depressive symptoms than in the control group; and other authors (Goldston et al., 2007; Wilson et al., 2009; Dahle et al., 2011) have reported similar findings.

Somatic complaints, and social and attention problems were also reportedly more common in our students with dyslexia than in their typically developing counterparts. The somatic complaints they described included migraines, headaches, stomach ache, nausea, vomiting, skin diseases, eye problems, tics, fatigue, and dizziness. Other authors also found that individuals with dyslexia reported experiencing psychophysiological symptoms and psychosomatic disorders such as headache and stomach ache (Willcutt and Pennington, 2000; Arnold et al., 2005). These findings may mean that students with dyslexia might be more prone to show anxiety and worries in the above-mentioned physical symptoms. An alternative explanation could be that undergraduates with dyslexia are able to identify psychological symptoms less easily than physiological symptoms. Either way, it is important to acknowledge the relatively small entity of such somatic effects.

Sabornie (1994), and Wiener and Schneider (2002) noted that young adults with dyslexia often report social problems, including difficult relationships with peers (such as fear of being ridiculed or unappreciated by others), loneliness, dependence on adults, jealousy, distress when speaking, and a preference for relationships with younger people. Findings from the present study confirm such social problems in the dyslexic group. This picture is also in line with a few reports on children with dyslexia (La Greca, 1981), or learning disabilities (LD). For instance, Nabuzoka and Smith (1993) found that children with LD were often ejected and very few were popular among their peers. These children were seen to be more shy, help-seeking, and more liable to bullying than non-LD children; only a few emerged as cooperative team members or leaders. That is why children with LD often receive training in social skills (see the meta-analysis by Forness and Kavale, 1996). As for attention problems, our dyslexic group mentioned more often than the control group that they had difficulty concentrating and sustaining their attention, suffered from mental confusion or daydreaming, and had a tendency to exhibit hyperactive behavior (e.g., they were unable to stay seated for long, or they were sometimes restless, impulsive, or irritable). Here again, these findings corroborate other authors’ reports of people with dyslexia having attention problems – especially in childhood – even when no ADHD has been diagnosed (Dykman and Ackerman, 1991; Heiervang et al., 2001; Arnold et al., 2005; Willcutt et al., 2007).

No differences emerged between our two groups in terms of resilience, mixed anxious/depressive features, thought problems, or rule-breaking and aggressive behavior. These data are in contrast with other reports of young adults with dyslexia having lower levels of resilience, more mental health problems, a greater probability of withdrawal from school, and more juvenile delinquency issues than typically developing young adults (Svetaz et al., 2000; Scanlon and Mellard, 2002; Ahrens et al., 2010; Nelson and Harwood, 2011). This discrepancy between the present results and findings reported elsewhere in the literature may be due to the different nature of the samples concerned: the young adults with dyslexia considered in the above-mentioned studies were not attending university like the participants in our sample. A direct comparison between our findings and those of previous studies on the psychological and psychopathological features of samples of young adults with dyslexia who were not continuing their formal education would be inappropriate because the latter probably lacked the opportunity to compensate for their reading and writing disabilities over time. Our participants also had by a number of protective factors, which may have limited the potential for psychological distress associated with dyslexia (Morrison and Cosden, 1997; Hellendoorn and Ruijssenaars, 2000; Al-Yagon and Mikulincer, 2004; Ingesson, 2007; Stampoltzis and Polychronopoulou, 2009; Pizzoli et al., 2011). For example, they had been diagnosed with dyslexia at an early age; 70% of them had received speech therapy at primary and/or secondary school, or had been helped by teachers to improve their reading and writing skills; they enjoyed an adequate social support, particularly from their families (especially their mothers), and from school teachers. It is also worth adding that our students with dyslexia were assessed at the start of their university careers, before taking any exams, so they did not have the chance to experience academic failure, and they consequently remained strongly motivated and had a positive attitude to their university careers. Such a positive attitude may have affected their self-perception - and their responses to the questionnaires as a result (though the same could be said of their typically developing counterparts).

There are some issues worth considering that may prevent us from generalizing the results of the present study. First, the sample sizes were small – reflecting the low percentage of adolescents with dyslexia who enroll at university. Second, it is not compulsory for university students to disclose their dyslexia; our sample represents a group of individuals with dyslexia who entered the study of their own free will and who were seeking help, so our results may not be fully generalizable to other university students with dyslexia. Third, since our data on the students’ socioemotional features rely exclusively on self-report measures, potentially useful information that might have been gained from past teachers or parents is lacking. Finally, published studies on the emotional well-being of undergraduate students refer mainly to the United States and the United Kingdom. Given the likely differences in the academic worlds of these countries, the findings emerging in one country cannot be generalized to others. In particular, under the Italian school system, children and adolescents with dyslexia usually attend mainstream schools where they follow a personalized education plan. This plan is a structured document outlining their academic goals, compensatory and dispensatory measures, assessments on their academic goals, and the need for some additional help, such as a longer time during exams. Children with dyslexia also often attend specific training sessions during their primary and secondary school years.

Despite the above-mentioned limitations, the present study also has two important strengths. First of all, it provides further evidence of the long-term psychological implications of dyslexia in a population that has been under-investigated to date. Second, to the best of our knowledge, the present findings represent a first attempt to explore the psychological issues of Italian university students with dyslexia. Our study may be one of the first to focus on exploring the socioemotional features of orthographically shallow language-speaking university students with dyslexia, since the literature available to date on this issue has almost exclusively concerned deep language-speaking undergraduates with dyslexia (e.g., Gregg et al., 1992; Hoy et al., 1997; Mattek and Wierzbicki, 1998; Riddick et al., 1999; Carroll and Iles, 2006; Nelson and Gregg, 2012).

## Conclusion

In the light of our findings and considerations, it is worth encouraging the provision of university services for dyslexics, to help them strengthen their reading and writing abilities, and prevent and/or manage any dyslexia-related psychological distress. In this perspective, future studies could also investigate other aspects relevant to this issue, such as the use of more specific self-report measures or clinical interviews to identify other important protective factors that might help young adults with dyslexia to cope with their disorder and its consequences. In addition to resilience, adaptive coping strategies, personal strengths, acceptance of the disability, self-efficacy, and personality features may be variables that would influence functional psychosocial adjustment.

Finally, conducting longitudinal studies would enable us to disentangle whether students whose dyslexia is diagnosed in childhood (and who therefore receive support early on) retain the strong psychological resilience seen in our sample as they progress through their university degree programs, and may experience failure. It would likewise be of interest to see whether students whose dyslexia is diagnosed late (at university) show less positive coping or adjustment strategies, and whether this profile persists even after they have received appropriate support to improve their study skills and strategies.

## Author Contributions

MG, IM project design and research supervisors; MG, GB introduction and discussion writing; AR participants recruitment and testing; GB, AR data analysis; SC method and results writing.

## Conflict of Interest Statement

The authors declare that the research was conducted in the absence of any commercial or financial relationships that could be construed as a potential conflict of interest.
